# High genetic diversity among extraintestinal *Escherichia coli* isolates in pullets and layers revealed by a longitudinal study

**DOI:** 10.1186/s12917-016-0859-5

**Published:** 2016-10-07

**Authors:** Surya Paudel, Beatrix Stessl, Claudia Hess, Angelika Zloch, Michael Hess

**Affiliations:** 1 Department for Farm Animals and Veterinary Public Health, Clinic for Poultry and Fish Medicine, University of Veterinary Medicine, Veterinärplatz 1, 1210 Vienna, Austria; 2 Department for Farm Animals and Veterinary Public Health, Institute of Milk Hygiene, Milk Technology and Food Science, University of Veterinary Medicine, Veterinärplatz 1, Vienna, 1210 Austria

**Keywords:** *Escherichia coli*, Longitudinal epidemiology, Pullets, Layers, Pulsed-field gel electrophoresis

## Abstract

**Background:**

Various information about the genetic diversity of *Escherichia coli* isolates from chickens are available but a detailed epidemiological investigation based upon isolates obtained from interrelated pullet and layer flocks is still missing. Therefore, in the course of a longitudinal epidemiological study on pullets and layers, 144 *E. coli* isolates from chickens with or without pathological lesions of the reproductive tract were serotyped and genotyped with pulsed-field gel electrophoresis (PFGE). These isolates were collected during rearing, peak and at the end of production. The actual study is the first of its kind so as to elucidate genetic relatedness among extraintestinal *E. coli* isolated from chickens with varying pathological conditions in interrelated layer farms/flocks at different stages of rearing.

**Results:**

Serotyping revealed that 63.19 % of the isolates could not be assigned to any of the three serotypes tested whereas 30.55 % of the isolates belonged to serotype O1:K1, 4.86 % to O2:K1 and 1.38 % to O78:K80. After macrorestriction digest with *Xba*I, 91.66 % of the isolates were typeable resulting in 96 distinct PFGE profiles. Among them, five PFGE types included isolates collected from diseased chickens as well as from birds without pathological lesions. This finding shows that pathogenicity of *E. coli* in layers seems to be largely influenced by concurrent susceptibility factors. Furthermore, in six out of eight cases where two isolates were collected from each of eight birds, different PFGE types were found in the same or different organs of the same bird. The existence of predominant or persistent *E. coli* genotypes was only observed in two cases.

**Conclusions:**

It is concluded that extraintestinal *E. coli* genotypes and serotypes in pullets and layers are heterogenous and also do not maintain a single clonality within the same bird. The facts that *E. coli* strains did not show any definite clonal population structure based on geographical region, age of the host and pathological lesions should have relevance in further epidemiological studies and control strategies.

## Background

*Escherichia coli* isolates that are extraintestinal in nature are associated with the disease named colibacillosis that can infect all aged groups of chickens [1]. In layers, the pathogen is able to cause a systemic infection leading to fibrinous polyserositis, pericarditis, perihepatitis, salpingitis, peritonitis, salpingoperitonitis and a decrease in egg production ultimately leading to severe economic losses [2–8]. Despite serological diversities, serogroups such as O1, O2 and O78 are mostly implicated in disease conditions [9–11]. Until now, the pathogenicity of *E. coli* infection in chickens is not well understood. Several putative virulence and virulence-associated genes have been reported in avian pathogenic *E. coli* (APEC) [1, 11, 12]. However, the fact that a single genetic trait cannot separate disease-associated *E. coli* from commensal intestinal isolates raised certain concern on the definition of APEC as a single pathotype [13, 14].

From an epidemiological point of view, understanding the clonal population structure of extraintestinal *E. coli* involving a longitudinal sampling scheme in interrelated rearing and laying flocks has a high priority. Thus we performed a longitudinal study in order to characterize the relatedness among *E. coli* isolates from systemic organs of pullets and layers kept in alternative housing systems in Austria. Beside the determination of the serotype, pulsed-field gel electrophoresis (PFGE) was applied for genetic fingerprinting which has higher discriminating power compared to other methods such as multilocus sequence typing [15]. PFGE is more applicable to investigate large-scale genomic diversity within a distinct population and has also been previously applied to infer molecular relatedness among APEC isolates in other geographical locations [16–18].

## Methods

### Flock history, sampling and *E. coli* isolation

The present investigation was focused on extraintestinal *E. coli* isolates from pullets and laying hens kept in alternative husbandry system that were located in different provincial states of Austria. Six rearing and eight related layer farms comprising 15 layer flocks were included in the longitudinal study. Rearing farms are designated with letter “R” along with farm numbers as R_I_ – R_VI_ (e. g. R_I_ is rearing farm 1). The layer flocks are designated with letter “L” along with the flock number and the corresponding rearing farm (e. g. L_1/I_ indicate for layer flock 1 that comes from rearing farm 1). Detailed information on farms and flocks is provided in Table 1.

**Table 1:** Farms and flocks included in the study

Rearing farm		Layer farm/flock				
Farm identification^a^	Location	Farm	Flock identification^b^	Location	Housing system^c^	Flock size^d^
R_I_	Lower Austria	1	L_1/I_	Styria	FR	7500
			L_2/I_	Styria	FR	3800
			L_3/I_	Styria	DL	3440
R_II_	Salzburg	2	L_1/II_	Carinthia	ORG	3000
		3	L_2/II_	Lower Austria	ORG	3000
R_III_	Styria	4	L_1/III_	Burgenland	ORG	6000
			L_2/III_	Burgenland	ORG	6000
			L_3/III_	Burgenland	ORG	6000
R_IV_	Upper Austria	5	L_1/IV_	Lower Austria	DL	5980
			L_2/IV_	Lower Austria	DL	10890
			L_3/IV_	Lower Austria	DL	9030
R_V_	Styria	6	L_1/V_	Styria	DL	17738
		7	L_2/V_	Styria	DL	14950
R_VI_	Burgenland	8	L_1/VI_	Styria	DL	2950
			L_2/VI_	Styria	FR	7300

^a^six rearing farms are indicated as R_I_ - R_VI_ (all birds were kept in deep litter system)

^b^layer flocks are designated with letter “L” along with flock number/corresponding rearing farm number

^c^housing system: FR – conventional free range, ORG – organic free range, DL – deep litter

^d^number of birds in each layer flock

**Fig. 1:**
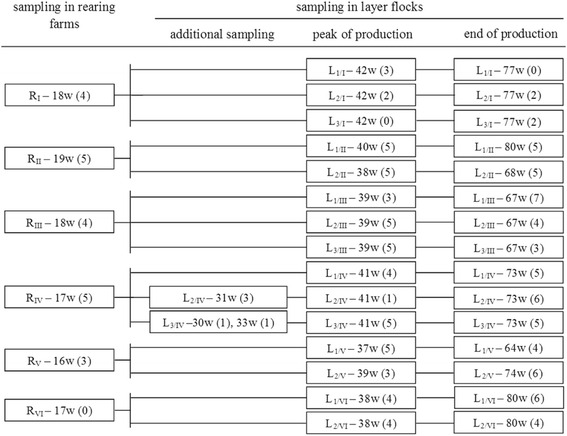
Sampling scheme for the longitudinal study of extraintestinal *Escherichia coli* in pullets and layers. Each box represents an individual sampling event and includes the following information: farm/flock identification (rearing farms are indicated as R_I_ - R_VI_ and layer flocks are designated with letter “L” along with flock number/corresponding rearing farm number) – age of birds during sampling (weeks) – number of *E. coli* isolates in parenthesis

In total, 188 birds were sampled for extraintestinal *E. coli* based on the sampling scheme as shown in Fig. 1. Sampling was performed during rearing (age of birds: 16–19 weeks), at the peak of production (age of birds: 37–42 weeks) and at the end of production (age of birds: 64–80 weeks). In each of the sampling events, five birds per rearing farm/layer flock were necropsied and sampled for extraintestinal *E. coli*. In two flocks of one layer farm (L_2/IV_ and L_3/IV_), additional samplings were included at 30–33 weeks of age (eight birds in total) because of increased mortality and drop in egg production. The sampling scheme was focused on the isolation of *E. coli* from the reproductive organs (ovary and oviduct). Where *E. coli* could not be isolated from the reproductive tract, isolates from liver, heart or lung were chosen for further investigation. For isolation of *E. coli*, organ samples were aseptically streaked on McConkey agar (Scharlau, Vienna, Austria) and incubated at 37 °C for 24 h aerobically. On the following day, subcultures were made on Columbia agar supplemented with 5 % sheep blood (COS agar, BioMérieux, Vienna, Austria) and incubated at 37 °C for 24 h aerobically. Most of the isolates were collected from ovary or oviduct (number of isolates *n =* 106) followed by liver (*n =* 25), lung (*n =* 10) and heart (*n =* 3). Details on *E. coli* isolates included in the present study are shown in Table 2. Isolates from rearing farms are marked with letter “R” along with farm number and bird number (e. g. R_I_-1 denotes for *E. coli* isolate collected from rearing farm 1 and bird number 1). Likewise, isolates from layer flocks are labelled with letter “L” along with flock number/corresponding rearing farm number – time of sampling (A: peak of production, B: end of production, Z1 or Z2: first or second additional samplings) – bird number – organs (only in those birds from where two samples were collected). For instance, L_1/I_-A-1 denotes for the isolate collected from layer flock 1 that originated from rearing farm 1; at the peak of production; bird number 1. Generally, one *E. coli* isolate per bird was included for further characterization. However, in the case of eight birds, two isolates per bird from the same or different organs were collected at the same sampling event: L_1/III_-B-2-ovary1, L_1/III_-B-2-ovary2; L_1/III_-B-3-ovary, L_1/III_-B-3-oviduct; L_1/III_-B-4-oviduct1, L_1/III_-B-4-oviduct2; L_2/IV_-B-1-ovary, L_2/IV_-B-1-liver; L_1/V_-A-4-oviduct, L_1/V_-A-4-ovary; L_2/V_-B-5-ovary, L_2/V_-B-5-heart, L_1/VI_-B-1-ovary, L_1/VI_-B-1-oviduct; L_2/VI_-B-2-ovary, L_2/VI_-B-2-oviduct.

**Table 2:** *E. coli* isolates and pathological findings in reproductive tract

Farm/flock	Isolate identification^a^	Age (weeks)	Reproductive lesions	Serotype	PFGE type
Rearing farm 1					
R_I_	R_I_-1	18	no	nt^b^	LA23
	R_I_-2		no	nt	nt
	R_I_-3		no	nt	LA20
	R_I_-4		no	nt	LA19
L_1/I_-A	L_1/I_-A-1	42	no	nt	S31
	L_1/I_-A-2		no	nt	S37
	L_1/I_-A-5		no	O78:K80	S6
L_2/I_-A	L_2/I_-A-1	42	no	O1:K1	S2
	L_2/I_-A-5		no	O1:K1	S15
L_2/I_-B	L_2/I_-B-4	77	oophoritis	nt	S22
	L_2/I_-B-5		oophoritis	nt	S23
L_3/I_-B	L_3/I_-B-1	77	oophoritis and salpingitis	O2:K1	S19
	L_3/I_-B-2		oophoritis	O2:K1	S19
Rearing farm 2					
R_II_	R_II_-1	19	no	O1:K1,O2:K1,O78:K80	Sa3
	R_II_-2		no	O1:K1,O2:K1,	Sa1
	R_II_-3		no	nt	Sa4
	R_II_-4		no	O1:K1	Sa5
	R_II_-5		no	nt	Sa2
L_1/II_-A	L_1/II_-A-1	40	no	O1:K1	nt
	L_1/II_-A-2		no	O1:K1	nt
	L_1/II_-A-3		no	O1:K1	nt
	L_1/II_-A-4		no	O1:K1	nt
	L_1/II_-A-5		no	O1:K1	nt
L_2/II_-A	L_2/II_-A-1	38	degeneration of oviduct	O1:K1,O2:K1,	nt
	L_2/II_-A-2		no	O1:K1,O2:K1,	LA10
	L_2/II_-A-3		oophoritis	O2:K1	LA6
	L_2/II_-A-4		oophoritis	O1:K1,O2:K1,O78:K80	LA9
	L_2/II_-A-5		oophoritis	O1:K1,O2:K1,	LA2
L_1/II_-B	L_1/II_-B-1	80	egg peritonitis	nt	Ca4
	L_1/II_-B-2		no	O2:K1	Ca2
	L_1/II_-B-3		egg peritonitis	nt	Ca3
	L_1/II_-B-4		egg peritonitis	nt	Ca5
	L_1/II_-B-5		egg peritonitis	nt	Ca1
L_2/II_-B	L_2/II_-B-1	68	oophoritis	nt	LA17
	L_2/II_-B-2		oophoritis	nt	LA18
	L_2/II_-B-3		oophoritis	nt	LA18
	L_2/II_-B-4		oophoritis	nt	LA7
	L_2/II_-B-5		oophoritis	nt	LA18
Rearing farm 3					
R_III_	R_III_-1	18	no	nt	S16
	R_III_-2		no	nt	S5
	R_III_-3		no	nt	S5
	R_III_-4		no	O1:K1,O2:K1,	S4
L_1/III_-A	L_1/III_-A-1	39	oophoritis	nt	B15
	L_1/III_-A-3		no	O1:K1	B14
	L_1/III_-A-5		oophoritis	nt	B3
L_2/III_-A	L_2/III_-A-1	39	no	nt	B4
	L_2/III_-A-2		no	O1:K1	B6
	L_2/III_-A-3		no	nt	B4
	L_2/III_-A-4		no	nt	B8
	L_2/III_-A-5		no	nt	B8
L_3/III_-A	L_3/III_-A-1	39	oophoritis	nt	B1
	L_3/III_-A-2		oophoritis	nt	B1
	L_3/III_-A-3		oophoritis	nt	B1
	L_3/III_-A-4		oophoritis	nt	B1
	L_3/III_-A-5		oophoritis	nt	B17
L_1/III_-B	L_1/III_-B-2-ovary1	67	egg peritonitis	O1:K1	B13
	L_1/III_-B-2-ovary2		egg peritonitis	nt	B12
	L_1/III_-B-3-ovary		no	O1:K1	B12
	L_1/III_-B-3-oviduct		no	nt	B12
	L_1/III_-B-4-oviduct1		no	nt	B5
	L_1/III_-B-4-oviduct2		no	nt	B11
	L_1/III_-B-5		degeneration of ovary and oviduct	nt	B10
L_2/III_-B	L_2/III_-B-1	67	no	O1:K1	B7
	L_2/III_-B-2		egg peritonitis	O1:K1	B7
	L_2/III_-B-3		degeneration of ovary and oviduct	O1:K1	B9
	L_2/III_-B-4		egg peritonitis	O1:K1	B9
L_3/III_-B	L_3/III_-B-3	67	no	O1:K1	B16
	L_3/III_-B-4		oophoritis	O1:K1	B2
	L_3/III_-B-5		no	O1:K1	nt
Rearing farm 4					
R_IV_	R_IV_-1	17	no	nt	Ua2
	R_IV_-2		no	nt	Ua1
	R_IV_-3		no	nt	Ua1
	R_IV_-4		no	nt	Ua3
	R_IV_-5		no	nt	Ua4
L_1/IV_-A	L_1/IV_-A-1	41	no	nt	LA16
	L_1/IV_-A-2		no	nt	LA15
	L_1/IV_-A-3		no	nt	LA15
	L_1/IV_-A-4		no	nt	LA27
L_2/IV_-A	L_2/IV_-A-2	41	no	O1:K1	LA13
L_3/IV_-A	L_3/IV_-A-1	41	degeneration of ovary and oviduct	nt	LA21
	L_3/IV_-A-2		egg peritonitis	nt	LA21
	L_3/IV_-A-3		degeneration of ovary and oviduct	nt	LA11
	L_3/IV_-A-4		degeneration of ovary and oviduct	nt	LA11
	L_3/IV_-A-5		no	nt	LA4
L_1/IV_-B	L_1/IV_-B-1	73	degeneration of ovary and oviduct	nt	LA25
	L_1/IV_-B-2		oophoritis	nt	LA1
	L_1/IV_-B-3		oophoritis	nt	nt
	L_1/IV_-B-4		oophoritis	nt	LA5
	L_1/IV_-B-5		oophoritis	nt	LA25
L_2/IV_-B	L_2/IV_-B-1-ovary	73	oophoritis	nt	LA25
	L_2/IV_-B-1-liver		oophoritis	O1:K1	LA25
	L_2/IV_-B-2		oophoritis	O1:K1	LA25
	L_2/IV_-B-3		oophoritis	O1:K1	LA26
	L_2/IV_-B-4		oophoritis	O1:K1	LA25
	L_2/IV_-B-5		oophoritis	nt	LA28
L_3/IV_-B	L_3/IV_-B-1	73	oophoritis	O1:K1	LA3
	L_3/IV_-B-2		oophoritis	O1:K1	LA14
	L_3/IV_-B-3		oophoritis	O1:K1	LA25
	L_3/IV_-B-4		oophoritis	nt	LA8
	L_3/IV_-B-5		oophoritis	O1:K1	LA25
L_2/IV_-Z1	L_2/IV_-Z1-1	31	oophoritis	O1:K1,O2:K1,O78:K80	LA22
	L_2/IV_-Z1-2		no	O1:K1,O2:K1,O78:K80	LA22
	L_2/IV_-Z1-3		egg peritonitis	O1:K1,O2:K1,O78:K80	LA22
L_3/IV_-Z1	L_3/IV_-Z1-2	30	degeneration of ovary and oviduct	O1:K1,O2:K1,	LA24
L_3/IV_-Z2	L_3/IV_-Z2-1	33	no	nt	LA12
Rearing farm 5					
R_V_	R_V_-3	16	no	O1:K1,O2:K1,	nt
	R_V_-4		no	O1:K1	S7
	R_V_-5		no	O1:K1	S7
L_1/V_-A	L_1/V_-A-2	37	no	O1:K1	nt
	L_1/V_-A-3-oviduct		no	nt	S1
	L_1/V_-A-4-oviduct		egg peritonitis	nt	S18
	L_1/V_-A-4-ovary		egg peritonitis	O1:K1	S7
	L_1/V_-A-6		egg peritonitis	O1:K1	nt
L_1/V_-B	L_1/V_-B-1	64	egg peritonitis	O2:K1	S8
	L_1/V_-B-2		egg peritonitis	O2:K1	S9
	L_1/V_-B-3		egg peritonitis	nt	S25
	L_1/V_-B-5		egg peritonitis	O2:K1	S9
L_2/V_-A	L_2/V_-A-3	39	degeneration of ovary	nt	S27
	L_2/V_-A-4		degeneration of ovary	O78:K80	S29
	L_2/V_-A-5		no	nt	S3
L_2/V_-B	L_2/V_-B-1	74	no	O1:K1	S10
	L_2/V_-B-2		no	O1:K1	S10
	L_2/V_-B-3		oophoritis and salpingitis	O1:K1	S10
	L_2/V_-B-4		oophoritis	nt	S20
	L_2/V_-B-5-ovary		egg peritonitis	O1:K1	S34
	L_2/V_-B-5-heart		egg peritonitis	O1:K1	S34
Rearing farm 6					
L_1/VI_-A	L_1/VI_-A-1	38	no	nt	S36
	L_1/VI_-A-2		no	nt	S21
	L_1/VI_-A-4		oophoritis	nt	S11
	L_1/VI_-A-5		no	nt	S21
L_1/VI_-B	L_1/VI_-B-1-ovary	80	degeneration of ovary and oviduct	O1:K1	S33
	L_1/VI_-B-1-oviduct		degeneration of ovary and oviduct	nt	S12
	L_1/VI_-B-2		degeneration of ovary and oviduct	O1:K1	S33
	L_1/VI_-B-3		oophoritis	O1:K1	S26
	L_1/VI_-B-4		degeneration of ovary and oviduct	nt	S24
	L_1/VI_-B-5		egg peritonitis	O1:K1	S35
L_2/VI_-A	L_2/VI_-A-1	38	no	nt	S17
	L_2/VI_-A-2		no	nt	S13
	L_2/VI_-A-4		oophoritis	nt	S32
	L_2/VI_-A-5		oophoritis, degeneration of oviduct	nt	S17
L_2/VI_-B	L_2/VI_-B-2-ovary	80	oophoritis	nt	S28
	L_2/VI_-B-2-oviduct		oophoritis	O1:K1	S30
	L_2/VI_-B-4		degeneration of ovary	nt	S14
	L_2/VI_-B-5		egg peritonitis	O1:K1	S32

Age of birds, lesions in the reproductive tract, serotypes and PFGE types of each *E. coli* isolates are provided in the corresponding vertical line

^a^isolates identification: isolates from rearing farms are marked with letter “R” along with farm number and bird number. Likewise, isolates from layer flocks are labelled with letter “L” along with flock number/corresponding rearing farm number – time of sampling (A: peak of production, B: end of production, Z1 or Z2: first or second additional samplings) – bird number – organs (only in those birds from where two samples were collected)

^b^non-typeable

Additionally, gross pathological lesions of the reproductive tract were recorded. Pullets from all six rearing farms did not show any gross pathological lesions. At the peak of production, some *E. coli* isolates originated from birds showing lesions in the reproductive tract, including egg peritonitis, inflammation of ovary and/or oviduct and degeneration of ovary and/or oviduct in 5, 17 and 7 birds respectively. Also, at the end of production, egg peritonitis, inflammation of ovary and/or oviduct and degeneration of ovary and/or oviduct were recorded in 15, 40 and 9 birds, respectively. In additional samplings, gross pathological lesions found in the reproductive tract comprised egg peritonitis in one bird, inflammation of ovary and/or oviduct in two birds and degeneration of ovary and oviduct in one bird.

### Subtyping of *E. coli* isolates

Serotyping was performed on 144 *E. coli* isolates applying a slide agglutination test to *Escherichia coli* O1:K1, O2:K1 and O78:K80 antisera following supplier’s guidelines (Animal Health and Veterinary Laboratory Agency, Weybridge, Surrey, UK).

For PFGE, *E. coli* isolates were grown on COS agar at 37 °C for 24 h. The plug preparation and PFGE was performed according to the standardized Pulsenet International protocol for *E. coli* O157:H7, *E. coli* non-O157, *Salmonella* serotypes, *Shigella sonnei* and *Shigella flexneri* (http://www.pulsenetinternational.org/assets/PulseNet/uploads/pfge/PNL05_Ec-Sal-ShigPFGEprotocol.pdf; accessed on 18.12.2015). The macrorestriction digest was performed applying *Xba*I (50 U/sample; Thermo Fisher Scientific, Fermentas; Waltham, Massachusetts, USA) at 37 °C for 2–3 h. Restricted samples were separated in a 1 % (w/v) SeaKem Gold agarose gel (Lonza Group AG, Basel, Switzerland) in 0.5 × TBE buffer at 6 V/cm on a Chef DR _II_I system (Bio-Rad Laboratories, Inc.). A linear ramping factor with pulse times from 2.2 to 54.2 s at 14 °C and an inclined angle of 120° was applied for 22.5 h. The gels were stained with ethidium bromide (Sigma Aldrich, Vienna, Austria), digitally photographed with Gel Doc 2000 (Bio-Rad Laboratories, Inc.) and normalized as TIFF images (BioNumerics 6.6 software Applied Math NV, Sint-Martens-Latem, Belgium) applying the PFGE global standard *Salmonella* ser. Braenderup H9812. In order to identify indistinguishable PFGE types, a Dice co-efficient similarity of 100 % was used.

### *E. coli* confirmation of non-typeable genotypes

Partial sequencing of 16S rRNA gene was done in PFGE non-typeable isolates (*n =* 12) as described previously [19]. For this purpose, strains were grown on COS agar plates at 37 °C for 24 h. DNA extraction was done from two to three colonies using DNeasy Blood and Tissue Kit (QIAGEN, Hilden, Germany) following manufacturer’s recommendation. PCR was performed with a set of primers: 16S F 5’-GGCGGCRKGCCTAAYACATGCAAGT-3’ and 16S R 5’-GACGACARCCATGCASCACCTGT-3’. Amplification was carried out in 25 μl reaction volume consisting of 12.5 μl of HotStarTaq Master Mix (Qiagen, Hilden, Germany), 8 μl of nuclease free distilled water, 1 μl of each forward and reverse primers (10pmol/μl) and 2.5 μl of DNA template. The PCR thermocycler was programmed as: initial denaturation at 95 °C for 15 min followed by 40 cycles of heat denaturation at 94 °C, annealing at 60 °C for 1 min and extension at 72 °C for 1.5 min. Final elongation was performed at 72 °C for 10 min. The PCR products were visualized by agarose gel electrophoresis. The gel slices were cut and purified using QIAquick® gel extraction kit (QIAGEN, Germany). Samples were then dispatched to LGC genomics GmbH (Berlin, Germany) for sequencing. The data obtained were processed with software Accelrys Gene v2.5 (Accelrys Inc) and analyzed with BLAST search in NCBI database.

### Antimicrobial resistance (AMR)

Sixteen *E. coli* isolates originating from eight birds (two isolates per bird from the same or different organs) were investigated for the potential difference in AMR among strains isolated from the same organ (2 birds) or from different organs of the same bird (6 birds). The antimicrobial susceptibility test was performed using the disk diffusion method on Mueller-Hinton Agar (BioMeriéux, Vienna, Austria) according to Bauer et al. [20]). The following antimicrobials were tested: aminopenicilline [amoxicillin and ampicillin (each 10 μg)], aminoglycoside [gentamicin (10 μg), neomycin (30 μg)], tetracyclines [tetracycline and doxycycline (each 30 μg)], co-trimoxazole [sulphamethoxazole and trimethoprim (25 μg)], macrolide (tylosin 30 μg), quinolone [oxolinic acid 2 μg, enrofloxacin (5 μg)], cephalosporine [ceftiofur (30 μg)], polymyxin [colistin (10 μg)] and aminocyclitol [spectinomycin (100 μg)]. Multidrug resistance (MDR) among avian *E. coli* was defined as resistance to three or more classes of antimicrobial agents.

## Results

### Subtyping of *E. coli* isolates

**Fig. 2:**
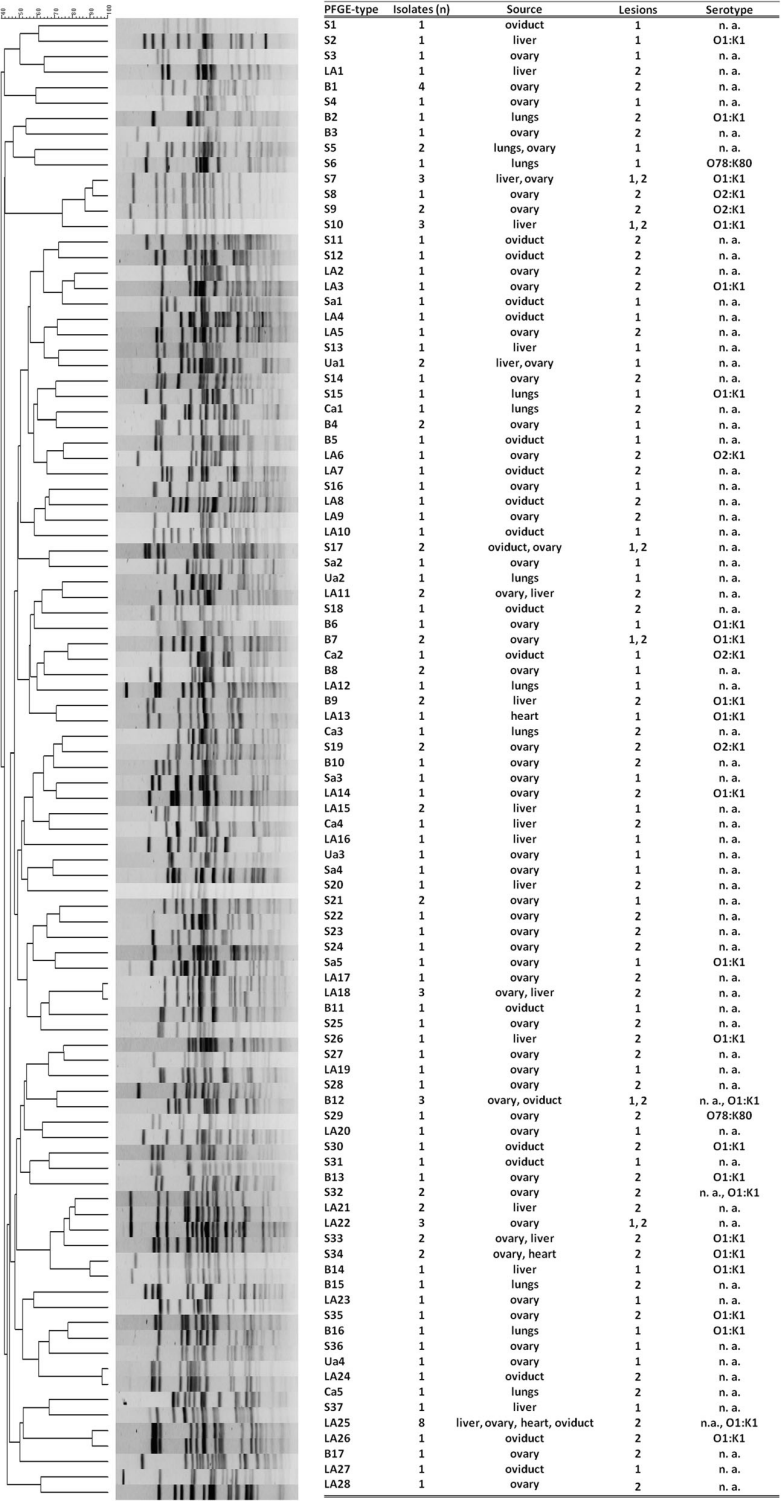
PFGE cluster analysis of *Escherichia coli* isolates from pullets and layers (restriction enzyme *Xba*I). The TIFF images were compared using BioNumerics 6.6 software (Applied Math NV, Sint-Martens-Latem, Belgium), and normalized using the PFGE global standard *Salmonella* ser. Braenderup H9812. Pattern clustering was performed using the unweighted pair group method using arithmetic averages (UPGMA) and the Dice correlation coefficient was applied with a position tolerance of 1.0 %. Information provided adjacent to the dendrogram include the PFGE-type in combination with areas of isolation (S: Styria, LA: Lower Austria, Ua: Upper Austria, Sa: Salzburg, B: Burgenland, Ca: Carinthia), number of isolates (n) in each PFGE type, organs of isolation (source) and serotype [not applicable (na) are untypeable isolates]. Furthermore, *E. coli* isolation was classified according to the absence (1) or presence (2) of lesions in the reproductive tract

Serotyping revealed that 44 isolates (30.55 %) were grouped as O1:K1 while 7 (4.86 %) and 2 (1.38 %) strains belonged to O2:K1 and O78:K80, respectively. Furthermore, 91 isolates (63.19 %) could not be assigned to a definite serotype using these three antisera as they did not show agglutination (*n =* 79) or reacted positive with more than one anti-serum used (*n =* 12). Isolates that did not show agglutination with any or reacted positive with more than one anti-serum were assigned as non-typeable (Table 2). The PFGE analysis of 132 *E. coli* isolates resulted in a heterogenous PFGE cluster: 96 *E. coli* profiles were obtained after macrorestriction digest applying *Xba*I while 12 isolates were non-typeable. The dendrogram obtained from the cluster analysis is shown in Fig. 2.

The most abundant *E. coli* PFGE-profile was LA25 (*n =* 8) which included strains from three layer flocks (L_1/IV_-B, L_2/IV_-B and L_3/IV_-B) that originated from a single rearing farm (R_IV_). All these isolates were associated with lesions in the reproductive tract. Likewise, B1 included four isolates from birds with inflammation of ovaries in the same flock (L_3/III_-A). Furthermore, *E. coli* genotypes which caused reproductive tract lesions in more than one laying bird at one sampling occasion from the same flock were: B9 (*n =* 2), LA11 (*n =* 2), LA18 (*n =* 3), LA21 (*n =* 2), S19 (*n =* 2), S33 (*n =* 2), S34 (*n =* 2) and S9 (*n =* 2).

Interestingly, *E. coli* genotypes B7 (*n =* 2), B12 (*n =* 3), LA22 (*n =* 3), S10 (*n =* 3) and S17 (*n =* 2) included isolates from both normal and diseased chickens. S5 (*n =* 2), Ua1 (*n =* 2), B4 (*n =* 2), B8 (*n =* 2), LA15 (*n =* 2) and S21 (*n =* 2) were present in pullets or layers without clinical signs. PFGE type S7 included three *E. coli* isolates that were collected from two pullets without pathological lesions and one laying hen with egg peritonitis originating from the same rearing farm.

### DNA sequencing

The non-typable isolates were confirmed by partial sequencing of 16S rRNA gene as *E. coli* (99-100 % identity). Accession numbers of the isolates to the European Nucleotide Archive are as follows: R_I-2_: LT548255, L_1/II_-A-1: LT548253, L_1/II_-A-2: LT548254, L_1/II_-A-3: LT548256, L_2/II_-A-1: LT548257, L_3/III_-B-5: LT548258, R_V_-3: LT548251, L_1/V_-A-2: LT548250, L_1/V_-A-6: LT548252. Following three isolates had 100 % identity with the existing database: L_1/II_-A-4: JQ975905.1, L_1/II_-A-5: JQ975905.1, L_1/IV_-B-3: KU560507.1.

### Antimicrobial resistance (AMR)

The results of antibiotic resistance tests are shown in Table 3. These *E. coli* isolates were considered for the test in order to investigate similarities or differences in antibiotic sensitivity profiles between two strains collected from the same bird. All isolates were resistant to tylosin. Additionally, MDR was observed in three isolates originating from different birds. Two of these were resistant to five antibiotic substances {aminopenicilline (amoxicillin and ampicillin), tetracycline, doxycycline, sulphamethoxazole and trimethoprim} and the other to three antibiotic substances (oxolinic acid, doxycycline and neomycin). The following pair of isolates had non-identical pattern of resistance towards several antimicrobials used: 1) L_1/III_-B-2-ovary1 and L_1/III_-B-2-ovary2: amoxicillin; 2) L_1/III_-B-3-ovary and L_1/III_-B-3-oviduct: ampicillin, amoxycillin, doxycycline, tetracycline and sulphamethoxazole + trimethoprim; 3) L_1/III_-B-4-oviduct1 and L_1/III_-B-4-oviduct2 : ampicillin, amoxycillin, doxycycline, tetracycline and sulphamethoxazole + trimethoprim; 4) L_2/IV_-B-1-ovary and L_2/IV_-B-1-liver : amoxicillin; 5) L_1/V_-A-4-oviduct and L_1/V_-A-4-ovary : oxolinic acid; 6) L_2/V_-B-5-ovary and L_2/V_-B-5-heart : doxycycline, enrofloxacin, neomycin; 7) L_1/VI_-B-1-ovary and L_1/VI_-B-1-oviduct : oxolinic acid; 8) L_2/VI_-B-2-ovary and L_2/VI_-B-2-oviduct : amoxicillin and doxycycline.

**Table 3:** Antibiotic resistance test of 16 *Escherichia coli* isolates collected from 8 birds (two isolates per bird)

Antibiotics	Isolates															
	L1/III-B-2-ovary1	L1/III-B-2-ovary2	L1/III-B-3-ovary	L1/III-B-3-oviduct	L1/III-B-4-oviduct1	L1/III-B-4-oviduct2	L2/IV-B-1-ovary	L2/IV-B-1-liver	L1/V-A-4-oviduct	L1/V-A-4-ovary	L2/V-B-5-ovary	L2/V-B-5-heart	L1/VI-B-1-ovary	L1/VI-B-1-oviduct	L2/VI-B-2-ovary	L2/VI-B-2-oviduct
Ampicillin	I	I	**I**	**R**	**R**	**S**	S	S	I	I	S	S	I	I	I	I
Amoxycillin	I	R	I	R	R	I	**S**	**I**	I	I	I	I	I	I	**R**	**I**
Ceftiofur	S	S	S	S	S	S	S	S	S	S	S	S	S	S	S	S
Colistin	S	S	S	S	S	S	S	S	S	S	S	S	S	S	S	S
Doxycycline	I	I	**S**	**R**	**R**	**S**	S	S	S	S	**S**	**R**	S	S	**I**	**S**
Enrofloxacin	S	S	S	S	S	S	S	S	S	S	**S**	**I**	S	S	S	S
Gentamicin	S	S	S	S	S	S	S	S	S	S	S	S	S	S	S	S
Neomycin	S	S	S	S	S	S	S	S	S	S	**S**	**R**	S	S	S	S
Oxolinic acid	S	S	S	S	S	S	S	S	**S**	**R**	R	R	**R**	**S**	S	S
Tetracycline	S	S	**S**	**R**	**R**	**S**	S	S	S	S	S	I	S	S	S	S
Tylosin	R	R	R	R	R	R	R	R	R	R	R	R	R	R	R	R
Spectinomycin	S	S	S	S	S	S	S	S	S	S	S	S	S	S	S	S
Sulphamethoxazole + trimethoprim	S	S	**S**	**R**	**R**	**S**	S	S	S	S	S	S	S	S	S	S

Each isolate ID is designated with letter “L” along with the number of layer flock and rearing farm – A (sampling at the peak of production) or B (sampling at the end of production) – number of sampled bird – organ of isolation – isolate number (in case when two isolates were collected from the same organ). Antibiotic resistance pattern of two isolates from the same bird are in bold letter and highlighted if they showed different sensitivity to antimicrobials used. S: sensitive, I intermediate, R: resistant

## Discussion

An infection with *E. coli* in layers is regarded as one of the major problems in global poultry industry that might cause reproductive disorders referred as salpingitis/peritonitis/salpingoperitonitis and peritonitis syndrome ultimately leading to severe economic losses on commercial farms [6]. In this regards, an epidemiological knowledge of the disease and disease causing agent is fundamental in order to develop effective control and prophylactic strategies. Here, we studied molecular epidemiology of *E. coli* isolates collected from pullets and layers in a longitudinal sampling study in Austria. Data obtained from genetic fingerprinting by PFGE were analyzed together with serotypes, geographical regions of isolation, and concurrent pathological lesions in each of the sampled birds.

In total, more than half of the *E. coli* isolates (*n =* 91/144) could not be assigned to a single serotype using antibodies against O1:K1, O2:K1 and O78:K80. Furthermore, for those isolates that could be assigned to one of the named serotypes, no correlation was found between a specific serotype and the occurrence of lesions in birds. In previous studies, it was also shown that *E. coli* isolates collected from diseased birds display a high serological diversity [16, 21, 22], demonstrating as high as 62 different O serogroups [21]. Thus classifying *E. coli* strains into a definite serotype might sometimes be somewhat challenging. Hence, our finding is in agreement with a previous notion that serotyping alone might not be helpful as a tool for characterization of *E. coli* [16].

In this study, the PFGE subtyping of *E. coli* isolates (*n =* 132) resulted in 96 *Xba*I profiles. Exclusively in two events, the same PFGE profile was seen in isolates from different sampling dates in mutually related farms/flocks, indicating potential *E. coli* persistence. The PFGE-type S7 (*n =* 3) included isolates from pullets (*n =* 2, rearing farm R_V_) without pathological lesions and from one layer in the corresponding flock L_1/V_ suffering from egg peritonitis and fibrinous oophoritis at the peak of production. In the second case, PFGE type S32 contained two isolates from the same layer flock (L_2/VI_) at the peak and end of production. One bird sampled at the peak of production showed inflammation of the ovary whereas egg peritonitis was diagnosed in the other birds necropsied at the end of production. These results indicate that some *E. coli* genotypes may retain in certain flocks at different stages of rearing but the associated pathological outcomes in birds can vary.

The genomic profile of extraintestinal *E. coli* with PFGE further revealed that strains collected from birds with pathological lesions can have 100 % genetic identity with strains that were collected from healthy birds. For instance, in PFGE type S10 (*n =* 3) in flock L_2/V_, two birds did not have any lesions while one had oophoritis and salpingitis. Likewise in PFGE type B7 (*n =* 2) in L_2/III_, one bird showed no lesions while in contrast, the other had egg peritonitis. Also, remaining isolates could not be grouped into distinct clonal clusters based on presence or absence of pathological lesions in sampled birds. This finding is in agreement with a previous study in broilers where authors have reported a high heterogenecity of *E. coli* isolates in broilers [13, 23]. It can be hypothesized that pathogenicity of extraintestinal *E. coli* in chickens is highly dependent on concurrent environmental and host susceptibility factors. Providing a suitable opportunity in certain circumstances, *E. coli* residing in clinically healthy chickens might turn up into pathogenic. The hypothesis is further supported by an earlier finding in broiler that many collibacillosis associated isolates might not be clearly distinguished solely on the basis of presence of virulence associated genes as compared to intestinal commensal *E. coli* [13].

In the present study, we found no evidence for clonality of *E. coli* with respect to geographical locations of farms. Previously, Ewers et al. (2004) found only a limited number of *E. coli* clones to be distributed in poultry production in Germany [16]. In another study, it was reported that chickens with peritonitis in a single flock were likely to be infected by the same *E. coli* strain [24]. Different to this, we did not find clonality of *E. coli* isolates in birds from the same flock showing gross pathological lesions in the reproductive tract thus maintaining a high heterogenicity of PFGE types. Interestingly, we further noticed that a single bird can harbour two different PFGE types of *E. coli* in the same or different organs. Thus, the study demonstrated that a layer can be infected simultaneously by different *E. coli* genotypes. A similar finding was previously reported in broilers [18]. However, in another study in layers, one PFGE type was found to be present in bone marrow of an individual bird [17]. It might be that in some organs *E. coli* isolates possess less or no genetic diversity due to an adaptation process, which should however be further elucidated. In the present study, we also tested antibiotic susceptibility of 16 isolates that were collected from eight birds. All the isolates were sensitive to ceftiofur, colistin, gentamicin and spectinomycin but the resistant rate to tylosin was found 100 %. Mixed results were obtained for other antibiotics tested. MDR was seen in 3/16 isolates showing resistance to as high as five different antibiotics used. Although the number of isolates included for antimicrobial susceptibility test in the actual study is not very high, it already provides an indication for the problem of antibiotic resistance in *E. coli* towards commonly used antimicrobials. In a recent report from China, *E. coli* isolates collected from chickens were sensitive to relatively newer antibiotics such as cephalosporin but MDR rate was as high as 80.25 % [11]. The results from the present study further indicate that isolates collected from the same bird may not necessarily have identical antibiotic sensitivity profiles. Thus it can be suggested that testing of the antibiotic sensitivity profile from just one isolate per bird might not be enough to decide the most appropriate treatment.

## Conclusions

Serotyping, antibiotic resistance test and genotypic fingerprinting of extraintestinal *E. coli* revealed that isolates exhibit high diversities within and between birds. As one bird can harbour different *E. coli* types an appropriate number of isolates should be considered for epidemiological studies and antibiotic sensitivity test.

## Abbreviations

AMR: Antimicrobial resistance
APEC: Avian Pathogenic *Escherichia coli*
COS: Columbia agar supplemented with 5 % sheep blood
MDR: Multidrug resistance.
PCR: Polymerase chain reaction
PFGE: Pulsed-field gel electrophoresis

## References

[1] Nolan LK, Barnes HJ, Vaillancourt JP, Abdul-Aziz T, Logue CM. Colibacillosis. In: Swayne DE, editor. Diseases of Poultry. 13th ed. Ames: John Wiley & Sons, Inc; 2013. p. 751–805

[2] Dhillon AS, Jack OK (1996). Two outbreaks of colibacillosis in commercial caged layers. Avian Dis.

[3] Vandekerchove D, De HP, Laevens H, Pasmans F (2004). Colibacillosis in caged layer hens: characteristics of the disease and the aetiological agent. Avian Pathol.

[4] Jordan FT, Williams NJ, Wattret A, Jones T (2005). Observations on salpingitis, peritonitis and salpingoperitonitis in a layer breeder flock. Vet Rec.

[5] Zanella A, Alborali GL, Bardotti M, Candotti P, Guadagnini PF, Martino PA, Stonfer M (2000). Severe *Escherichia coli* O111 septicaemia and polyserositis in hens at the start of lay. Avian Pathol.

[6] Landman WJ, van Eck JH (2015). The incidence and economic impact of the *Escherichia coli* peritonitis syndrome in Dutch poultry farming. Avian Pathol.

[7] Olsen RH, Bisgaard M, Christensen JP, Kabell S, Christensen H (2016). Pathology and Molecular Characterization of *Escherichia Coli* associated With the Avian Salpingitis-Peritonitis Disease Syndrome. Avian Dis.

[8] Srinivasan P, Balasubramaniam GA, Murthy TR, Balachandran P (2013). Bacteriological and pathological studies of egg peritonitis in commercial layer chicken in Namakkal area. Asian Pac J Trop Biomed.

[9] Dho-Moulin M, Fairbrother JM (1999). Avian pathogenic *Escherichia coli* (APEC). Vet Res.

[10] Ewers C, Jannsen T, Wieler LH. Avian pathogenic Escherichia coli (APEC). Berl Munch Tierarztl Wochenschr. 2003;116:381–95. 14526468

[11] Dou X, Gong J, Han X, Xu M, Shen H, Zhang D, Huang L, Liu J, Zou J (2016). Characterization of avian pathogenic *Escherichia coli* isolated in eastern China. Gene.

[12] Paixao AC, Ferreira AC, Fontes M, Themudo P, Albuquerque T, Soares MC, Fevereiro M, Martins L, de Sa MI C (2016). Detection of virulence-associated genes in pathogenic and commensal avian *Escherichia coli* isolates. Poult Sci.

[13] Kemmett K, Humphrey T, Rushton S, Close A, Wigley P, Williams NJ (2013). A longitudinal study simultaneously exploring the carriage of APEC virulence associated genes and the molecular epidemiology of faecal and systemic *E. coli* in commercial broiler chickens. PLoS One.

[14] Collingwood C, Kemmett K, Williams N, Wigley P (2014). Is the concept of Avian Pathogenic *Escherichia coli* (APEC) as a single pathotype is fundamentally flawed?. Frontiers in Veterinary Science.

[15] Torpdahl M, Skov MN, Sandvang D, Baggesen DL (2005). Genotypic characterization of *Salmonella* by multilocus sequence typing, pulsed-field gel electrophoresis and amplified fragment length polymorphism. J Microbiol Methods.

[16] Ewers C, Janssen T, Kiessling S, Philipp HC, Wieler LH (2004). Molecular epidemiology of avian pathogenic *Escherichia coli* (APEC) isolated from colisepticemia in poultry. Vet Microbiol.

[17] Landman WJ, Buter GJ, Dijkman R, van Eck JH (2014). Molecular typing of avian pathogenic *Escherichia coli* colonies originating from outbreaks of *E. coli* peritonitis syndrome in chicken flocks. Avian Pathol.

[18] Barbieri NL, de Oliveira AL, Tejkowski TM, Pavanelo DB, Matter LB, Pinheiro SR, Vaz TM, Nolan LK, Logue CM, de Brito BG, Horn F (2015). Molecular characterization and clonal relationships among *Escherichia coli* strains isolated from broiler chickens with colisepticemia. Foodborne Pathog Dis.

[19] Carroll NM, Jaeger EE, Choudhury S, Dunlop AA, Matheson MM, Adamson P, Okhravi N, Lightman S (2000). Detection of and discrimination between gram-positive and gram-negative bacteria in intraocular samples by using nested PCR. J Clin Microbiol.

[20] Bauer AW, Kirby WM, Sherris JC, Turck M (1966). Antibiotic susceptibility testing by a standardized single disk method. Am J Clin Pathol.

[21] Blanco JE, Blanco M, Mora A, Jansen WH, Garcia V, Vazquez ML, Blanco J (1998). Serotypes of *Escherichia coli* isolated from septicaemic chickens in Galicia (northwest Spain). Vet Microbiol.

[22] Zhao S, Maurer JJ, Hubert S, De Villena JF, McDermott PF, Meng J, Ayers S, English L, White DG (2005). Antimicrobial susceptibility and molecular characterization of avian pathogenic Escherichia coli isolates. Vet Microbiol.

[23] Pasquali F, Lucchi A, Braggio S, Giovanardi D, Franchini A, Stonfer M, Manfreda G (2015). Genetic diversity of *Escherichia coli* isolates of animal and environmental origins from an integrated poultry production chain. Vet Microbiol.

[24] Trampel DW, Wannemuehler Y, Nolan LK (2007). Characterization of *Escherichia coli* isolates from peritonitis lesions in commercial laying hens. Avian Dis.

